# Plasma glucose levels and diabetes are independent predictors for mortality in patients with COVID-19

**DOI:** 10.1017/S095026882200022X

**Published:** 2022-05-16

**Authors:** Hui Long, Jiachen Li, Rui Li, Haiyang Zhang, Honghan Ge, Hui Zeng, Xi Chen, Qingbin Lu, Wanli Jiang, Haolong Zeng, Tianle Che, Xiaolei Ye, Liqun Fang, Ying Qin, Qiang Wang, Qingming Wu, Hao Li, Wei Liu

**Affiliations:** 1 Tianyou Hospital, Wuhan University of Science and Technology, Wuhan, Hubei, P. R. China; 2 State Key Laboratory of Pathogen and Biosecurity, Beijing Institute of Microbiology and Epidemiology, Beijing, P. R. China; 3 Department of Healthcare, School of Health Sciences, Wuhan University, 115 Donghu Road, Wuhan, Hubei 430071, P. R. China; 4 Global Health Institute, Wuhan University, 8 South Donghu Road, Wuhan, Hubei 430072, P. R. China; 5 Center for Disease Control and Prevention of Central Theater Command, Beijing, Shijingshan District, China; 6 Department of Thoracic and Vascular Surgery, Wuhan No. 1 Hospital, Tongji Medical College, Huazhong University of Science and Technology, Wuhan 430022, P. R. China; 7 Department of Laboratorial Science and Technology, School of Public Health, Peking University, Beijing, P. R. China; 8 Department of Thoracic Surgery, Renmin Hospital of Wuhan University, Wuhan 430060, P. R. China; 9 Department of Laboratory Medicine, Tongji Hospital, Tongji Medical College, Huazhong University of Science and Technology, Wuhan, P. R. China; 10 Division of Infectious Disease, Key Laboratory of Surveillance and Early Warning on Infectious Disease, Chinese Center for Disease Control and Prevention, Beijing, P. R. China; 11 Institute of Infection, Immunology and Tumor Microenvironment, Hubei Province Key Laboratory of Occupational Hazard Identification and Control, Medical College, Wuhan University of Science and Technology, Wuhan 430065, P. R. China

**Keywords:** COVID-19, diabetes, hyperglycaemia, prognosis, risk factors

## Abstract

This study is performed to figure out how the presence of diabetes affects the infection, progression and prognosis of 2019 novel coronavirus disease (COVID-19), and the effective therapy that can treat the diabetes-complicated patients with COVID-19. A multicentre study was performed in four hospitals. COVID-19 patients with diabetes mellitus (DM) or hyperglycaemia were compared with those without these conditions and matched by propensity score matching for their clinical progress and outcome. Totally, 2444 confirmed COVID-19 patients were recruited, from whom 336 had DM. Compared to 1344 non-DM patients with age and sex matched, DM-COVID-19 patients had significantly higher rates of intensive care unit entrance (12.43% *vs.* 6.58%, *P* = 0.014), kidney failure (9.20% *vs.* 4.05%, *P* = 0.027) and mortality (25.00% *vs.* 18.15%, *P* < 0.001). Age and sex-stratified comparison revealed increased susceptibility to COVID-19 only from females with DM. For either non-DM or DM group, hyperglycaemia was associated with adverse outcomes, featured by higher rates of severe pneumonia and mortality, in comparison with non-hyperglycaemia. This was accompanied by significantly altered laboratory indicators including lymphocyte and neutrophil percentage, C-reactive protein and urea nitrogen level, all with correlation coefficients >0.35. Both diabetes and hyperglycaemia were independently associated with adverse prognosis of COVID-19, with hazard ratios of 10.41 and 3.58, respectively.

## Introduction

The World Health Organization recently declared the outbreak of 2019 novel coronavirus pneumonia (COVID-19) a global pandemic. Increased morbidity and mortality are particularly seen in older persons and those presenting with comorbidities such as overt diabetes, obesity and hypertension [1–3]. Among all the listed comorbidities, diabetes mellitus (DM) is the leading predictor for mortality and morbidity in COVID-19 [4, 5]. Of major clinical importance, studies have so far reported a two-to-threefold higher prevalence of diabetes in patients with severe infections requiring admission to intensive care units (ICUs) and invasive ventilation compared with those with less severe infection, as well as an increased mortality rate at least double in patients with diabetes [4, 6, 7]. Two of the largest series of diabetic patients with COVID-19 were reported in New York City that included 5700 patients, which however, only mentioned that, of the patients who died, those with diabetes were more likely to have received invasive mechanical ventilation or care in the ICU compared with those without diabetes, with no further information provided regarding the characteristics of diabetes patients [8]. Another study performed in China based on preexisting diabetes patients, revealed that people in hyperglycaemia status have a higher mortality compared to those in non-hyperglycaemia status [9].

However, a high degree of heterogeneity is observed in diabetes population, such as type of diabetes, quality of glucose control, duration of disease as well as diabetic complications all show diversity. One key question is the effect on poorer outcomes because of DM or the hyperglycaemia cannot be separated, as COVID-19 can cause secondary hyperglycaemia as revealed in previous studies [9, 10]. The multiple complications associated with diabetes, especially hypertension, cardiovascular disease or renal disorders, were frequently encountered [11]. How these comorbidities can interact and modify the direct effect from DM was not determined. Furthermore, the potential effect of frequently used medications for diabetes patients in affecting the COVID-19 disease, such as renin–angiotensin–aldosterone system blockers, insulin, dipeptidyl peptidase-4 inhibitors are also disputed [12–15]. On the other hand, it remains uncertain whether people with diabetes are at higher risk of infection, or merely due to the compound effect from old age [16]. Here, we performed a matched case–control study to figure out how the presence of diabetes affects the infection, progression and prognosis of COVID-19, and to explore the effective therapy that can treat the diabetes-complicated patients with COVID-19.

## Materials and methods

### Patients and methods

A multicentre study was performed in four hospitals in Wuhan between 1st January and 31st March 2020, i.e. Tianyou Hospital of Wuhan University of Science and Technology, Wuhan First Hospital, Renmin Hospital of Wuhan University and Tongji Hospital, Tongji Medical College. All are the major tertiary hospitals in Wuhan and responsible for the treatments for COVID-19 assigned by the government. COVID-19 was diagnosed as positive for severe acute respiratory syndrome coronavirus 2 (SARS-CoV-2) by real-time reverse transcriptase-polymerase chain reaction assay of oropharyngeal swab specimens [17]. This study was approved by the Institutional Ethics Board of each participating hospital, respectively. The electronic medical records were reviewed by a trained group of medical care workers to extract data on demographic feature, medical history, clinical symptoms and signs on admission, blood test for haematology and biochemistry, as well as the treatment measures that were consecutively applied during hospitalisation. Clinical outcomes were acquired from all recruited patients, with the primary outcome defined as death or survived, and the second outcome defined as ICU entrance, multiple organ failure, septic shock and acute respiratory distress syndrome (ARDS).

### Definitions of DM

We defined illness onset as the first day of reported symptoms consistent with COVID-19. According to ADA's criteria for the diagnosis of diabetes [18], patients were classified as having DM if there was a documented medical history of DM and/or with a haemoglobin A1c (HbA1c) level >48 mmol/mol (HbA1c >6.5%). Patients with random glucose readings >180 mg/dl during hospitalisation, but with no documented medical history of DM, as well as at a normal HbA1c level (≤6.5%), were defined as hyperglycaemia. The severity of COVID-19 was assessed during hospitalisation and classified as mild, moderate, severe or critical, according to the COVID-19 management guidance [17], based on which severe and critically ill subtypes were grouped into severe COVID-19 and the others were used as non-severe COVID-19.

### Statistical analysis

Categorical variables were expressed as frequency and percentages (%), and continuous variables were expressed as mean (standard deviation, s.d) if they are normally distributed or median (inter-quartile range, IQR) if not. Categorical variables between groups were analysed using Student's *t* test or Mann–Whitney *U* test as appropriate. Mean values and percentages were compared among different groups by analysis of variance and *χ*^2^ tests, as appropriate. For comparison of clinical symptoms, laboratory measurements and clinical outcomes between DM and non-DM, we applied propensity score matching (PSM) to adjust sex and age using the nearest-neighbour matching method by R package ‘MatchIt’. PSM refers to the expression of the influence of multiple covariables by the change of propensity score (PS) value, matching between groups according to PS value and finally analysing the effect value within the matching group with balanced distribution of covariables [19]. The Cox proportional hazards regression model was used to assess the association between glucose levels and COVID-19-related mortality. The survival days of each case were used as the dependent variable and the age, sex and days from onset to admission to hospital, preexisting comorbidities and usage of drugs, the presence of DM and status of glucose control were included as independent variables. To avoid potential heterogeneity from different data sources, we also included the hospitals that treated patients as covariates. To demonstrate any interaction effect between age and DM on the risk of death, we additionally created an interactive item of DM and age in the model. All statistical analyses were performed using R software 3.6.3 version. A *P* value of <0.05 was considered statistically significant.

## Results

### Baseline characteristics of COVID-19 patients with DM and their comparison with general DM patients

During the study period, a total of 2444 patients were enrolled in our analysis (median age: 61, IQR: 47–70 years). Of these 2444 patients, 35.72% were men and 35.9% were >65 years old. The median duration from symptoms onset to hospital admission and from hospitalisation to death were 8 (IQR: 5, 14) and 7 (3, 13) days, respectively. Based on criteria, 336 patients had DM, including DM with hypertension (*n* = 197), DM with coronary heart disease (CHD) (*n* = 52) and DM with cerebrovascular disease (CVD) (*n* = 26). Older age (65 *vs.* 60) rather than sex (31.55% *vs.* 35.86% of male) was associated with the presence of DM (Table 1). The comparison between all DM and non-DM patients disclosed significant higher presence of four commonly seen clinical symptoms/syndromes (dyspnoea, shortness of breath, confusion and coma) in the DM patients (Supplementary Table S1). Of totally 41 laboratory evaluations that were compared, 14 showed significant difference between two groups, which might be partially due to the older age or more comorbidities in the DM group (Supplementary Table S1). A significantly higher fatality rate was observed from the DM patients (25.00% *vs.* 13.99%, *P* < 0.001) (Table 1).

**Table 1.** Comparison of demographic characteristics and clinical outcome between COVID-19 patients with or without DM

	Total (*N* = 2444)	DM (*n* = 336)	Non-DM (*n* = 1994)	Matched non-DM (*n* = 1344)	*P*-value^a^	*P*-value^b^
Demographic characteristics						
Sex – male	873 (35.72)	106 (31.55)	715 (35.86)	426 (31.70)	0.142	1
Age (median, IQR)	61 (47, 70)	65 (57, 74)	60 (45.25, 69)	65 (57, 73)	<0.001	0.417
Onset to admission (days)	8 (5, 14)	8 (4, 14)	8 (5, 14)	8 (5, 14)	0.539	0.277
Admission to death (days)	7 (3, 13)	7 (3, 12)	6 (3, 13)	6 (3, 13)	0.774	0.615
Comorbidities						
Hypertension	772 (31.59)	197 (58.63)	571 (28.64)	464 (34.52)	<0.001	<0.001
CHD	197 (8.06)	52 (15.48)	145 (7.27)	130 (9.67)	<0.001	0.003
CVD	106 (4.34)	26 (7.73)	80 (4.01)	71 (5.28)	0.003	0.127
Glycaemic control status						
Non-hyperglycaemia	871 (35.64)	80 (23.81)	726 (36.41)	517 (42.34)	<0.001	<0.001
Hyperglycaemia	167 (6.83)	75 (22.32)	86 (4.31)	67 (2.53)		
Clinical severity						
Non-severe COVID-19	1664 (68.09)	194 (57.74)	1376 (69.01)	480 (35.71)	<0.001	0.031
Severe COVID-19	780 (31.91)	142 (42.26)	618 (30.99)	864 (64.29)		
Adverse clinical events						
ICU	79 (5.59)	23 (12.43)	55 (5.23)	45 (6.58)	<0.001	0.014
Heart failure	33 (4.27)	11 (15.58)	21 (5.38)	19 (8.00)	0.009	0.103
Kidney failure	22 (2.85)	8 (9.20)	14 (2.90)	12 (4.05)	0.011	0.027
Respiratory failure	131 (16.97)	30 (31.91)	97 (19.56)	85 (27.60)	0.011	0.355
Liver failure	7 (0.91)	1 (1.43)	6 (1.56)	4 (1.71)	1	1
Multiple organ failure	23 (2.98)	7 (9.72)	14 (3.62)	11 (4.68)	0.032	0.148
Septic shock	18 (2.33)	5 (5.81)	13 (2.71)	11 (3.73)	0.171	0.371
ARDS	26 (3.37)	7 (11.67)	19 (6.01)	14 (6.73)	0.159	0.272
Clinical outcome^c^						
Cured and discharged	1937 (79.26)	234 (69.64)	1613 (80.89)	1023 (76.12)	<0.001	<0.001
Death	369 (15.1)	84 (25.00)	279 (13.99)	244 (18.15)		

DM, diabetes mellitus.

a Comparison between DM-COVID-19 patients and all non-DM-COVID-19 patients.

b Comparison between DM-COVID-19 patients and matched non-DM-COVID-19 patients.

c Cured/discharged cases and deaths were not total at 2444 because there were 138 cases were transferred to other hospitals and their outcomes were untraceable.

For patients without a history of DM, we redefined DM in terms of HbA1c levels (pre-DM: 5.7% < HbA1c ≤ 6.5%; DM: HbA1c > 6.5%). HbA1c was measured in a total of 99 patients, 66 of whom had no history of DM. Of the 66 patients, 21 was classified as normal group, 20 had pre-DM and 25 had DM. DM patients were more likely to have hyperglycaemia and pre-DM patients also had a higher risk of hyperglycaemia than those with normal HbA1c (44.0% *vs.* 25.0% *vs.* 9.5%, *P* = 0.032). However, due to the small sample size, we did not find significant differences in other characteristics and clinical outcomes among the three groups (Supplementary Table S4).

Based on a most recent national surveillance data [20], we found a significantly higher proportion of self-reported diabetes in the COVID-19 patients than that of the general population (12.7% *vs.* 6%, *P* < 0.001), this overrepresentation was also observed in the 18–29, 40–49 and ≥70 years old, and for male and female separately. In contrast, a highly comparable proportion of DM was observed between COVID-19 patients and that of the general population (13.7% *vs.* 12.8%), and a lower proportion was observed in COVID-19 patients of 50–59, 60–69 and ≥70 age groups. More DM was found in the female while less DM was found in the male for the COVID-19 patients than the general population (Table 2).

**Table 2.** Proportion of DM in the recruited COVID-19 patients *vs*. in the general population

	COVID-19 patients (*N* = 2444)		The general population (*n* = 75 880)			
	Self-reported diabetes	DM	Self-reported diabetes	*P*-value^a^	DM^b^	*P*-value^c^
Overall	311 (12.7%)	336 (13.7%)	4553 (6%)	<0.001	9682 (12.8%)	0.45
Age group^d^						
18–29	3 (2.9%)	3 (2.9%)	143 (0.8%)	<0.001	358 (2.0%)	0.45
30–39	8 (3.0%)	8 (3.0%)	393 (2.6%)	0.80	951 (6.3%)	0.06
40–49	28 (8.2%)	31 (9.1%)	801 (4.8%)	0.006	2020 (12.1%)	0.15
50–59	56 (12.3%)	62 (13.6%)	1351 (10.6%)	0.28	2688 (21.1%)	<0.001
60–69	88 (13.4%)	99 (15.1%)	1223 (14.9%)	0.34	2364 (28.8%)	<0.001
≥70	128 (20.9%)	133 (21.7%)	875 (16.5%)	0.008	1685 (31.8%)	<0.001
Sex						
Male	94 (10.8%)	106 (12.1%)	2357 (6.4%)	<0.001	5058 (13.7%)	0.01
Female	217 (13.8%)	230 (14.64%)	2188 (5.6%)	<0.001	4624 (11.8%)	<0.001

DM, diabetes mellitus.

a Comparison of the proportion of self-reported diabetes between COVID-19 patients and that of general population.

b DM was defined as described in the Method section.

c Comparison of the proportion of DM between COVID-19 patients and that of general population.

d Patients <18 years old were excluded from the current analysis.

### Comparison between COVID-19 patients with or without DM patients matched by age and sex

To attain comparability between the DM and non-DM patients, we selected 1344 non-DM patients with age and sex matched by PSM. The coexistence of other medical conditions was still higher in the DM group than the non-DM, including more hypertension and CHD (*P* < 0.001 and *P* = 0.002, respectively; Table 1). Most of clinical manifestations were different between DM *vs.* non-DM after the matching, except for coma (Supplementary Table S1). The number of laboratory evaluations that was significantly different between two groups was reduced to nine (Supplementary Table S1). As expected, the DM patients had higher levels of random plasma glucose (8.02 *vs.* 5.69) and HbA1c (8 *vs.* 5.9) (Supplementary Table S1).

The complications of COVID-19, including ICU entrance and the development of organ failure were compared, among which ICU entrance and kidney failure were observed with higher frequency in the DM than matched non-DM patients (12.43% *vs.* 6.58%, *P* = 0.014; 9.20% *vs.* 4.05%, *P* = 0.027). Significantly higher mortality rate was observed from the DM patients than the matched non-DM COVID-19 patients (25.00% *vs.* 18.15%, *P* < 0.001) (Table 1).

We made additional analysis on the COVID-19 patients with isolated DM and those with other comorbidities apart from DM. The DM–hypertension as the most frequently seen coexisting condition, showing no significant effect on more severe COVID-19 disease or any more adverse outcomes, when compared with isolated DM COVID-19 patients, and after adjusting the effect from age and sex (Supplementary Table S2). Other coexisting conditions, including DM–CVD (*N* = 3), and DM–coronary disease (*N* = 8) were not further analysed due to the small sample size.

### Hyperglycaemia is correlated with a higher risk of adverse outcome

To explore the effect from glycaemic status on the clinical outcome of DM-COVID-19 patients, we made comparison among four groups of patients that were matched for age and sex with PSM. It is disclosed that for both non-DM and DM groups, hyperglycaemia seemed to be associated with adverse clinical outcomes. In the non-DM group, hyperglycaemia featured by the higher presence of severe pneumonia, respiratory failure and mortality rate (all *P* < 0.005), in comparison with those with non-hyperglycaemia (Table 3). While the patients with hyperglycaemia with or without DM were highly comparable for the frequency of the adverse clinical outcome, and the mortality rate (Table 3). A similar result was disclosed by performing Cox proportional hazards regression analysis, in that DM showed the highest risk of death, with the hazard ratio (HR) estimated to be 10.41 (95% confidence interval (CI) 4.59–31.84; *P* < 0.001; Table 4), followed by the effect from hyperglycaemia (HR 3.58, 95% CI 1.87–6.86; *P* < 0.001). A significant negative interaction was observed between age and DM for the effect on death, with an HR of 0.95 (95% CI 0.91–0.98; *P* < 0.001), which indicated that the effect from simultaneous presence of older age and DM is lower than the sum of the isolated effect from older age and DM. This could be attributed to their overlapped effect on death. The delayed hospitalisation was related to mortality, with the HR < 1, indicating the shorter delay, the lower risk of fatal outcome. Prescription of glucocorticoid was also associated with significantly increased risk of death (HR 2.28, 95% CI 1.12–4.64; *P* = 0.023) (Table 4).

**Table 3.** Clinical outcomes of COVID-19 patients with or without DM stratified by the glucose control status

Clinical outcome		Non-DM patients^a^			DM patients^a^			
	Total (*N* = 580)	Non-hyperglycaemia (*n* = 344)	Hyperglycaemia^b^ (*n* = 86)	*P*-value	Non-hyperglycaemia (*n* = 75)	Hyperglycaemia^b^ (*n* = 75)	*P*-value	*P*-value^c^
Severity of pneumonia								
Non-severe	306 (52.76)	212 (61.63)	19 (22.09)	0.001	46 (61.33)	29 (38.67)	0.009	0.034
Severe	274 (47.24)	132 (38.37)	67 (77.91)		29 (38.67)	46 (61.33)		
Severity complications								
Heart failure	21 (3.62)	7 (8.86)	6 (17.14)	0.214	1 (6.25)	7 (21.88)	0.240	0.760
Kidney failure	13 (2.24)	4 (3.48)	3 (8.57)	0.355	1 (4.00)	5 (14.71)	0.228	0.477
Respiratory failure	73 (12.59)	25 (21.01)	27 (69.23)	<0.001	5 (19.23)	16 (42.11)	0.064	0.022
Liver failure	3 (0.52)	1 (1.28)	1 (3.03)	0.508	0 (0)	1 (3.33)	1	1
Multiple organ failure	15 (2.59)	5 (6.33)	4 (12.12)	0.445	3 (17.65)	3 (9.68)	0.651	1
Septic shock	11 (1.90)	4 (3.45)	3 (8.57)	0.354	1 (4.00)	3 (9.09)	0.627	1
ARDS	14 (2.41)	3 (4.11)	5 (15.63)	0.054	2 (12.50)	4 (14.81)	1	1
Outcomes								
Cured and discharged	418 (72.07)	287 (85.16)	36 (41.86)	<0.001	62 (82.67)	33 (44.00)	<0.001	0.522
Death	146 (25.17)	50 (14.84)	50 (58.14)		9 (12.00)	37 (49.33)		

a PSM was used to match age and sex for comparison between patients with non-hyperglycaemia groups within DM and non-DM groups, respectively.

b Hyperglycaemia indicated patients with random glucose readings >180 mg/dl during hospitalisation.

c *P* was compared between DM *vs*. non-DM patients with hyperglycaemia DM.

**Table 4.** Association between demography and drug use with fatal outcomes in patients with COVID-19 by univariable and multivariable Cox regression analyses

Variables	Patients^a^	HR			
	(*N* = 646)	Univariate (95% CI)	*P*-value	Multivariate (95% CI)	*P*-value
Sex-female	328 (50.77)	0.72 (0.45–1.15)	0.165	NS	
Age	60 (57, 69)	1.07 (1.05–1.09)	<0.001	1.07 (1.05–1.10)	<0.001
DM	112 (17.34)	2.14 (1.29–3.57)	0.003	10.41 (4.59–31.84)	<0.001
Interactive item (DM and age)^b^	–	1.01 (1.01–1.02)	<0.001	0.95 (0.91–0.98)	<0.001
Hyperglycaemia	88 (13.62)	7.29 (4.59–11.58)	<0.001	3.58 (1.87–6.86)	<0.001
Delayed hospitalisation^c^	9 (5, 14)	0.94 (0.91–0.97)	<0.001	0.97 (0.95–0.99)	0.001
Comorbidities					
Hypertension	184 (28.48)	2.04 (1.28–3.24)	<0.001	1.06 (0.63–1.80)	0.82
CHD	31 (4.80)	2.97 (1.52–5.81)	0.001	0.38 (0.14–1.08)	0.67
CVD	30 (4.64)	2.15 (1.02–4.50)	0.043	0.57 (0.23–1.41)	0.23
Drug use					
Antihypertensive drugs	220 (34.06)	1.61 (1.02–2.56)	0.042	1.34 (0.79–2.26)	0.27
Hypoglycaemic drugs^d^	80 (12.38)	3.28 (1.82–5.97)	<0.001	2.51 (1.35–4.64)	0.004
Glucocorticoid	226 (34.98)	6.84 (3.81–12.27)	<0.001	2.28 (1.12–4.64)	0.023
Antibiotic	534 (82.66)	6.78 (1.66–27.66)	0.008	2.66 (0.60-11.88)	0.20

The regression was conducted based on 646 COVID-19 cases (72 deaths included). The outcome in the model was death and the survival days of cases were used as dependent variable.

The hospitals were used as covariates to adjust for heterogeneity from different data sources and their results are not shown in the table.

NS, the variable has no significance and is excluded from final model.

a Categorical variables were expressed as frequency rates and percentages (%), and continuous variables were expressed as median (inter-quartile range, IQR).

b The interaction of DM and age was included as covariate in the Cox regression.

c The days from onset of COVID-19 to hospital admission.

d Hypoglycaemic drugs included insulin, metformin and sulphonylureas.

To explore the laboratory indicators that might mediate the association between glucose and adverse clinical outcome, we made a correlation analysis between the longitudinal glucose level and the other commonly seen laboratory indicators. The glucose level was inversely correlated with the median value of lymphocyte percentage, while positively correlated with those of HbA1c, C-reactive protein (CRP), percentage of neutrophils and urea nitrogen, all with correlation coefficients over 0.35 with significance (*P* < 0.001). The glucose level was also correlated with laboratory measurements that were indicative of liver function, renal function, inflammation responses and hypercoagulable state, which may contribute to a poorer prognosis of COVID-19, however, with the correlation coefficients less than 0.35 (Supplementary Table S3).

## Discussion

Diabetes is one of the known documented contributing host-related risk factor in severe COVID-19 and people with diabetes developing COVID-19 are at increased risk for complications and mortality [21, 22]. Although there were numerous studies showing that pre-existing diabetes is significantly associated with poorer outcome [4, 7], few of the studies have examined the impact of diabetes on COVID-19 severity independent of age, sex and other comorbidities, importantly hypertension. Here, we have examined the association between diabetes and severity of COVID-19 illness after considering the effect from these factors, based on which both diabetes and hyperglycaemia were independently associated with adverse outcome. Stratified analysis disclosed that patients without DM and hyperglycaemia had similar mortality and adverse complication as those of DM patients without hyperglycaemia. Likewise, non-diabetic patients with hyperglycaemia were similarly associated with higher risk of severe disease as the DM patients with hyperglycaemia. This conclusion was attained by using matching methods and multivariate analysis, and the robustness of the association between glycaemic variability and mortality was further confirmed in the Cox regression model showing an HR of 3.58.

The impact of hyperglycaemia on the pathogenesis of viral-induced respiratory diseases had been implicated from the perspective of increasing the glucose concentration in airway epithelial secretion and disrupting the defensive capacity of airway epithelia [23], as well as increasing the risk of severe hypoglycaemia [24]. This might underpin the currently observed increased occurrence of severe pneumonia, and respiratory failure in COVID-19 patients with hyperglycaemia, regardless of the DM status. This provided further evidence supporting the controlling of blood glucose as one of the priority treatment choice to improve the clinical outcome of the COVID-19 patients.

Diabetes has posed as an important public health problem in China, with highest risk outweighing that of all the other Asian countries [25]. Considering its high prevalence in China [26, 27] and frequent report among COVID-19 patients [28, 29], there is urgent need to solve the query whether patients with diabetes are at increased risk of COVID-19, which has been frequently explored yet with flaws of mixed population. For example, a large observational report including 116 COVID-19 patients indicated that DM was present with significantly higher frequency than that in the general population (12.5% *vs.* 7.1%) [30]. A retrospective study in Hangzhou revealed that among the 43 COVID-19 patients, 7.0% of them had diabetes, also higher than that of the control group [31]. However, all these studies only take into account self-reported DM in general population, which is a severe underestimation of the actual prevalence, in comparison with COVID-19 patients who undergo a completed physical examination on their admission into the hospital.

In China, the surveillance data of diabetes in the general population [20] have made this detailed comparison based on different definition criteria of diabetes possible. Here, we noticed highly differential results when the DM diagnosis was applied by different criteria in general population. When both the self-report diabetes plus abnormal glucose level was considered, we even observed no higher proportions of diabetes among COVID-19 patients than that of the general population aged >30 years old. Interestingly, we indeed observed more DM in the COVID-19 patients than the general population for female, while not for male. This result is rather credible considering the age-standardised prevalence of diabetes was significantly higher among men than among women (*P* < 0.001) [32]. Taken this together, it is highly possible that female with DM was indeed susceptible to SARS-CoV-2 infection. Still, the complex difference in major determining risk factors of diabetes and COVID-19, according to sex and age group [33, 34], had made the conclusion hard to be drawn for sure.

We also noted that when DM-COVID-19 patients were matched for age and sex, similar symptoms were observed as those without DM and their initial manifestation could be mild. However, significant DM *vs.* non-DM difference of the in-hospital laboratory abnormalities were identified during the hospitalisation that might be related to the adverse prognosis of DM-COVID-19, including elevated creatinine, urea nitrogen, uric acid and procalcitonin, four of them were indicative of kidney injury. This finding was consistent with the higher frequency of kidney failure in DM-COVID-19, a significant contributor to the fatal outcome. Multiple complications, especially renal disorders, were frequently encountered in DM patients [4]. As has been proven, COVID-19 may lead to renal injury and impair renal function [35], which was further aggravated by the DM-related renal comorbidities, leading to a worsened outcome.

A different panel of laboratory indictors was correlated with the glycaemic abnormalities, especially lymphopoenia, increased CRP, neutrophils percentage, all being inflammation-related biomarkers, indicating the degree of inflammation and possible secondary bacterial infection exacerbating COVID-19 [4, 36, 37]. This result supported the assumption that individuals with diabetes are at higher odds of infection resulting from multiple perturbations of innate immunity and also due to the deleterious role of hyperglycaemia on immune responses and defense against infections. It is generally believed that patients with two or more comorbidities might significantly escalate risks of poor prognosis compared with those who had a single comorbidity [38]. Unexpectedly, the current patients with diabetes and combined hypertension were not related to more severe illness in this study. Considering that these patients were older than those with isolated hypertension, and no severe disease was observed when age was adjusted, we suggested that previous significant findings from compound DM-hypertension was likely to be due to older age, instead of the disease itself. It was also previously known that an increase in systolic blood pressure is a protective factor against the death of these patients [39], thus corroborating the credibility of the current findings.

There are specific concerns as to the usage of steroids in patients with COVID-19. This remains controversial among numerous studies, although a very recent randomised controlled trial study provided evidence that dexamethasone is effective for severe patients with COVID-19 [40–42]. In our cohort, more glucocorticoid usage was associated with increased mortality after considering the effect from DM. However, no causality can be inferred under the context of the case–control study design, since the association might simply reflect the increased drug prescription in the DM-COVID-19 patients.

In summary, we found that both a known history of diabetes and glycaemic status during illness were independently predictive of COVID-19-related mortality. We were able to disclose that hyperglycaemia with or without DM during the hospitalisation were independent predictors for morbidity. Normalisation of blood glucose levels might be critical in reducing mortality and morbidity in both diabetic patients and non-diabetic patients. Intensive glucose monitoring and insulin therapy to obtain optimal metabolic control may improve the outcome of COVID-19 patients. These findings have the potential to provide therapeutic insights and contribute to precision medical interventions for COVID-19.

## Data Availability

Data are available from the corresponding author Wei Liu under reasonable request.

## Supplementary material

For supplementary material accompanying this paper visit https://doi.org/10.1017/S095026882200022X. click here to view supplementary material
