# Supplementary material for: Plasma glucose levels and diabetes are independent predictors for mortality in patients with COVID-19
Source: Epidemiol Infect. 2022 May 16;150:e106. doi: 10.1017/S095026882200022X (PMC9171060; doi:10.1017/S095026882200022X)
Supplement: Supplementary file 1 [file S095026882200022Xsup001.docx]

**Supplementary Material**

**Plasma glucose levels and diabetes are independent predictors for**

**mortality in patients with COVID-19**

Hui Long^1#^, Jiachen Li^2#^, Rui Li^3,4#^, Haiyang Zhang^2,5#^, Honghan Ge^2^, Hui Zeng^1^, Xi Chen^6^, Qingbin Lu^7^, Wanli Jiang^8^, Haolong Zeng^9^, Tianle Che^2^, Xiaolei Ye^2^, Liqun Fang^2^, Ying Qin^10*^, Qiang Wang^11*^, Qingming Wu^11*^, Hao Li^2*^, Wei Liu^2,7*^

^1^Tianyou Hospital, Wuhan University of Science and Technology, Wuhan, Hubei, P. R. China

^2^State Key Laboratory of Pathogen and Biosecurity, Beijing Institute of Microbiology and Epidemiology, Beijing, P. R. China

^3^Department of Healthcare, School of Health Sciences, Wuhan University, 115 Donghu Road, Wuhan, Hubei 430071, P. R. China

^4^Global Health Institute, Wuhan University, 8 South Donghu Road, Wuhan, Hubei 430072, P. R. China

^5^Center for Disease Control and Prevention of Central Theater Command, Shijingshan District, Beijing, China

^6^Department of Thoracic and Vascular Surgery, Wuhan No. 1 Hospital, Tongji Medical College, Huazhong University of Science and Technology, Wuhan 430022, P. R. China

^7^Department of Laboratorial Science and Technology, School of Public Health, Peking University, Beijing, P. R. China

^8^Department of Thoracic Surgery, Renmin Hospital of Wuhan University, Wuhan 430060, P. R. China

^9^Department of Laboratory Medicine, Tongji Hospital, Tongji Medical College, Huazhong University of Science and Technology, Wuhan, P. R. China

^10^Division of Infectious disease, Key Laboratory of Surveillance and Early Warning on Infectious Disease, Chinese Center for Disease Control and Prevention, Beijing, P. R. China

^11^Institute of Infection, Immunology and Tumor Microenvironment, Hubei Province Key Laboratory of Occupational Hazard Identification and Control, Medical College, Wuhan University of Science and Technology, Wuhan 430065, P. R. China# These authors contributed equally to this work.

***Correspondence:**

Y. Qin, Division of Infectious disease, Key Laboratory of Surveillance and Early Warning on Infectious Disease, Chinese Center for Disease Control and Prevention, Beijing, P. R. China. Email

: qinying@chinacdc.cn;

W. Liu and H. Li, State Key Laboratory of Pathogen and Biosecurity, 20 Dong-Da Street, Beijing Institute of Microbiology and Epidemiology, Fengtai District, Beijing, China 100071 Email: lwbime@163.com or liuwei@bmi.ac.cn; and lihao_1986@126.com

QM Wu, and Q Wang, Institute of Infection, Immunology and Tumor Microenvironent, Hubei Province Key Laboratory of Occupational Hazard Identification and Control, Medical College, Wuhan University of Science and Technology, Wuhan 430065, P.R.China. Email: wuhe9224@sina.com; and wangqiang@wust.edu.cn.

**Table S1. The clinical symptoms and laboratory measurements of COVID-19 patients with significant difference between DM vs. non-DM**

|  | **TOTAL**  **(N=2444)** | **Non-DM**  **(N=1994)** | **DM**  **(N=336)** | **Matched Non-DM**  **(N=1334)** | **P-value#** | **P-value*** |
| --- | --- | --- | --- | --- | --- | --- |
| **Selected Clinical symptoms** | | | | | | |
| Days of cough (median, IQR) | 16 (8, 24) | 16 (9, 26) | 12 (7, 21) | 17 (11, 25) | 0.026 | 0.091 |
| Dyspnea | 153 (6.26) | 114 (5.72) | 35 (10.42) | 87 (6.52) | 0.002 | 0.156 |
| Anhelation | 574 (23.49) | 451 (22.62) | 104 (30.95) | 342 (25.74) | 0.001 | 0.053 |
| Confusion | 71 (2.91) | 50 (2.51) | 19 (5.65) | 39 (2.92) | 0.008 | 0.053 |
| Coma | 69 (2.82) | 47 (2.36) | 20 (5.95) | 39 (2.92) | 0.003 | 0.033 |
|  | | | | | | |
| ALB, median g/L (IQR) | 37 (33.8, 40.5) | 37.1 (33.8, 40.5) | 36.55 (33, 39.65) | 36.5 (33, 39.5) | 0.044 | 0.947 |
| ALT, median U/L (IQR) | 25 (17.5, 39) | 25.05 (17.5, 40) | 21.50 (16, 33.88) | 25 (18, 37.35) | 0.001 | 0.005 |
| AST, median U/L (IQR) | 25.23 (20, 36) | 26 (20, 37) | 24 (18, 34) | 26 (20.38, 36.85) | 0.006 | 0.007 |
| CKMB, median μg (IQR) | 0.91 (0.45, 2.2) | 0.91 (0.41, 2.15) | 1.1 (0.64, 3.55) | 1.06 (0.55, 2.58) | 0.047 | 0.493 |
| Creatinine, median umol/L (IQR) | 64 (53, 78.45) | 64 (53.08, 77) | 70.3 (54.43, 92) | 62.9 (52.13, 76.38) | <0.001 | <0.001 |
| C-reactive protein  , median mg/L (IQR) | 12.56 (2.87, 34.36) | 12.48 (3.01, 34.78) | 20.28 (3.33, 44.85) | 15.45 (3.11, 39.1) | 0.001 | 0.045 |
| D-dimer, median mg/L (IQR) | 0.55 (0.25, 2.75) | 0.55 (0.24, 1.93) | 0.87 (0.39, 3.56) | 0.6 (0.27, 2.05) | <0.001 | 0.006 |
| Blood glucose, median mmol/L (IQR) | 5.75 (5.1, 7.1) | 5.64 (5.05, 6.69) | 8.02 (6.05, 11.65) | 5.69 (5.1, 6.74) | <0.001 | <0.001 |
| HbA1C, median % (IQR) | 6.5 (5.9, 8.45) | 5.85 (5.3, 6) | 8 (6.8, 9.6) | 5.9 (5.3, 6) | <0.001 | <0.001 |
| INR, median (IQR) | 1.04 (0.96, 1.11) | 1.03 (0.96, 1.11) | 1.05 (0.93, 1.13) | 1.04 (0.96, 1.12) | 0.047 | 0.131 |
| PT, median s (IQR) | 12.2 (11.4, 13.1) | 12.2 (11.34, 13.1) | 12.5 (11.6, 13.4) | 12.2 (11.4, 13.2) | 0.014 | 0.075 |
| RBC, median10^12^/L (IQR) | 4.12 (3.77, 4.52) | 4.14 (3.80, 4.53) | 3.99 (3.64, 4.37) | 4.01 (3.7, 4.38) | <0.001 | 0.460 |
| Ua, median μmmol/L (IQR) | 267.6 (209.5, 339) | 264 (205, 331) | 290.5 (224.5, 369.5) | 253 (201, 324.5) | 0.001 | <0.001 |
| UREA, median mmol/L (IQR) | 4.2 (3.35, 5.65) | 4.1, (3.3, 5.5) | 5.1 (3.77, 8.2) | 4.35 (3.5, 5.7) | <0.001 | <0.001 |

# the comparison between DM-COVID-19 patients and all Non-DM-COVID-19 patients* the comparison between DM-COVID-19 patients and matched Non-DM-COVID-19 patients

ALB: albumin; ALP: alkaline phosphatase; ALT: alanine aminotransferase; APTT: activated partial thromboplastin time; AST: Aspartate aminotransferase; CKMB: Creatine phosphokinase isoenzyme; FDP: fibrin degradation product; FIB: fibrinogen; GGT: Gamma Glutamyl Transpeptidase; GLB: globulin; IBIL: indirect bilirubin; INR: international normalized ratio; PLT: platelet; PCT: procalcitonin; RBC: red blood cell; TBIL: total bilirubin; TP: total protein; TT: thrombin time; Ua: uric acid; UREA: urea nitrogen; WBC: white blood cell.

**Table S2. Demographic characteristics and Clinical findings of COVID-19 patients with Isolated DM vs.DM-hypertension**

|  | **Isolated DM** | **DM-Hypertension** | **P-value** |
| --- | --- | --- | --- |
| **Demographic characteristics** |  |  |  |
| Sex-Male | 42 | 61 | 1 |
| Age (median, IQR) | 62 (53, 70) | 69 (60, 76) | <0.01 |
| **Symptoms** |  |  |  |
| Temperature (median, IQR) | 36.75 (36.3, 37.5) | 36.8 (36.3, 37.6) | 0.902 |
| Pulse (median, IQR) | 85.5 (77.25, 100) | 85 (75.5, 100) | 0.846 |
| Breath (median, IQR) | 20 (19, 22) | 20 (19, 22) | 0.535 |
| Systolic blood pressure (mmHg, median, IQR) | 128 (119, 133) | 133 (121, 140) | 0.057 |
| Diastolic blood pressure (mmHg, median, IQR) | 72.5 (70, 81.25) | 80 (70, 84) | 0.260 |
| Cough | 100 | 128 | 0.091 |
| Days of cough (median, IQR) | 10 (7, 15) | 15 (8, 21.5) | 0.114 |
| Expectoration | 15 | 27 | 0.211 |
| Dyspnea | 10 | 25 | 0.034 |
| Runny noses | 1 | 1 | 1 |
| Anhelation | 38 | 65 | 0.413 |
| Cyanosis of lips | 2 | 4 | 0.676 |
| Apathy | 0 | 2 | 0.495 |
| Irritability | 1 | 10 | 0.047 |
| Drowsiness | 1 | 5 | 0.403 |
| Confusion | 8 | 10 | 1 |
| Coma | 7 | 13 | 0.634 |
| Fever | 104 | 141 | 0.362 |
| Chills | 2 | 5 | 0.434 |
| Headache | 0 | 5 | 0.083 |
| Dizziness | 2 | 4 | 0.687 |
| Fatigue | 43 | 61 | 0.989 |
| Muscle | 1 | 5 | 0.214 |
| Arthralgia | 0 | 0 | 1 |
| Lymphadenectasis | 0 | 0 | 1 |
| Pharyngalgia | 3 | 1 | 0.517 |
| Pulmonary infections | 35 | 36 | 0.860 |
| Bronchitis | 0 | 1 | 1 |
| Splenauxe | 0 | 0 | 1 |
| **Comorbidities** |  |  |  |
| Hypertension | 0 | 197 | <0.001 |
| Coronary heart disease | 11 | 41 | 0.002 |
| Cerebrovascular disease | 6 | 20 | 0.079 |
| **Glycemic control** |  |  |  |
| Non-hyperglycemia | 45 | 35 | 0.259 |
| Hyperglycemia | 34 | 40 |  |
| **Severity of pneumonia** |  |  |  |
| Non severe COVID-19 | 83 | 110 | 0.452 |
| Severe COVID-19 | 54 | 87 |  |
| **Adverse clinical events** |  |  |  |
| ICU | 5 | 18 | 0.026 |
| Heart failure | 1 | 10 | 0.019 |
| Kidney failure | 1 | 7 | 0.069 |
| Respiratory failure | 7 | 22 | 0.007 |
| Liver failure | 0 | 1 | 1 |
| Multiple organ failure | 1 | 6 | 0.116 |
| Septic shock | 0 | 5 | 0.061 |
| Acute Respiratory Distress Syndrome | 2 | 5 | 0.247 |
| **Outcome** |  |  |  |
| Improved/Cured | 99 | 134 | 0.492 |
| Death/ uncured | 31 | 52 |  |

**Table S3. Correlation between blood glucose and commonly seen laboratory abnormalities in the COVID-19 patients**

| Variable | Correlation coefficient | p-value |
| --- | --- | --- |
| Glycated hemoglobin | 0.60 | <0.001 |
| C-reactive protein | 0.42 | <0.001 |
| Neutrophil percentage | 0.39 | <0.001 |
| UREA | 0.36 | <0.001 |
| CKMB | 0.35 | <0.001 |
| Neutrophil | 0.35 | <0.001 |
| D-dimer | 0.29 | <0.001 |
| INR | 0.29 | <0.001 |
| WBC | 0.29 | <0.001 |
| Prothrombin time | 0.28 | <0.001 |
| Direct bilirubin | 0.27 | <0.001 |
| FDP | 0.25 | <0.001 |
| PCT | 0.24 | <0.001 |
| AST | 0.21 | <0.001 |
| GGT | 0.2 | <0.001 |
| creatinine | 0.19 | <0.001 |
| ALP | 0.18 | <0.001 |
| FIB | 0.17 | <0.001 |
| APTT | 0.15 | <0.001 |
| GLB | 0.15 | <0.001 |
| TBIL | 0.15 | <0.001 |
| TT | 0.14 | <0.001 |
| ALT | 0.09 | 0.001 |
| Monocyte | 0.06 | 0.142 |
| Ua | 0.06 | 0.016 |
| IBIL | 0.04 | 0.236 |
| RBC | -0.03 | 0.325 |
| K^+^ | -0.05 | 0.052 |
| TP | -0.05 | 0.048 |
| Basophil | -0.08 | 0.061 |
| Ca^+^ | -0.12 | <0.001 |
| Eosinophil | -0.13 | 0.002 |
| Na^+^ | -0.17 | <0.001 |
| Monocyte ratio | -0.22 | <0.001 |
| PLT | -0.22 | <0.001 |
| Percent of eosinophil | -0.23 | <0.001 |
| ALB | -0.24 | <0.001 |
| Percent of basophils | -0.26 | <0.001 |
| Lymphocyte | -0.29 | <0.001 |
| Lymphocyte percentage | -0.38 | <0.001 |

ALB: albumin; ALP: alkaline phosphatase; ALT: alanine aminotransferase; APTT: activated partial thromboplastin time; AST: Aspartate aminotransferase; CKMB: Creatine phosphokinase isoenzyme; FDP: fibrin degradation product; FIB: fibrinogen; GGT: Gamma Glutamyl Transpeptidase; GLB: globulin; IBIL: indirect bilirubin; INR: international normalized ratio; PLT: platelet; PCT: procalcitonin; RBC: red blood cell; TBIL: total bilirubin; TP: total protein; TT: thrombin time; Ua: uric acid; UREA: urea nitrogen; WBC: white blood cell.

**Table S4. Comparison of demographic characteristics and outcome between HbA1c status in COVID-19 patients without history of DM**

|  | **Normal (n=21)** | **Pre-DM (n=20)** | **DM (n=25)** | **p-value**# |
| --- | --- | --- | --- | --- |
| **Demographic characteristics** |  |  |  |  |
| Sex-Male | 10 (47.6) | 8 (40.0) | 12 (48.0) | 0.842 |
| Age (median, IQR) | 48.0 (40.00, 69.00) | 59.00 (51.50, 70.75) | 61.00 (51.00, 68.00) | 0.224 |
| Onset to admission (days) | 8.00 (7.00, 11.50) | 10.00 (5.75, 15.00) | 7.00 (3.00, 20.00) | 0.787 |
| **Comorbidities** |  |  |  |  |
| Hypertension | 4 (19.0) | 7 (35.0) | 11 (44.0) | 0.199 |
| Cerebrovascular disease | 1 (4.8) | 2 (10.0) | 1 (4.0) | 0.672 |
| **Glycemic control status** |  |  |  |  |
| Non-hyperglycemia | 19 (90.5) | 15 (75.0) | 14 (56.0) | 0.032 |
| Hyperglycemia | 2 (9.5) | 5 (25.0) | 11 (44.0) |  |
| **Clinical severity** |  |  |  |  |
| Non severe COVID-19 | 18 (85.7) | 18 (90.0) | 22 (88.0) | 0.915 |
| Severe COVID-19 | 3 (14.3) | 2 (10.0) | 3 (12.0) |  |
| **Adverse clinical events** |  |  |  |  |
| ICU | 14 (66.7) | 11 (55.0) | 9 (36.0) | 0.779 |
| Heart failure | 1 (4.76) | 0 (0.0) | 0 (0.0) | 0.393 |
| Kidney failure | 0 (0.0) | 1 (5.0) | 0 (0.0) | 0.274 |
| Respiratory failure | 4 (19.0) | 1 (5.0) | 2 (8.0) | 0.316 |
| Liver failure | 1 (4.8) | 0 (0.0) | 0 (0.0) | 0.393 |
| Multiple organ failure | 0 (0.0) | 1 (5.0) | 0 (0.0) | 0.274 |
| Septic shock | 0 (0.0) | 0 (0.0) | 1 (4.0) | 0.393 |
| Acute Respiratory Distress Syndrome | 0 (0.0) | 0 (0.0) | 1 (4.0) | 0.393 |
| **Clinical outcome**^†^ |  |  |  |  |
| Cured and discharged | 17 (81.0) | 17 (85.0) | 21 (84.0) | 0.935 |
| Death | 4 (19.0) | 3 (15.0) | 4 (16.0) |  |
| # the comparison between normal patients, preDM-COVID-19 patients and DM-COVID-19 patients  Normal: HbA1c ≤ 5.7%; PreDM: 5.7 % < HbA1c ≤6.5%; DM: HbA1c > 6.5%. | | | | |
